# Treatment adherence and perception in patients on maintenance hemodialysis: a cross – sectional study from Palestine

**DOI:** 10.1186/s12882-017-0598-2

**Published:** 2017-05-30

**Authors:** Karam Sh. Naalweh, Mohammad A. Barakat, Moutaz W. Sweileh, Samah W. Al-Jabi, Waleed M. Sweileh, Sa’ed H. Zyoud

**Affiliations:** 1 Department of Medicine, College of Medicine and Health Sciences, An-Najah National University, Nablus, 44839 Palestine; 2 Division of Clinical and Community Pharmacy, Department of Pharmacy, College of Medicine and Health Sciences, An-Najah National University, Nablus, 44839 Palestine; 3 Department of Pharmacology and Toxicology, College of Medicine and Health Sciences, An-Najah National University, Nablus, 44839 Palestine; 4 Poison Control and Drug Information Center (PCDIC), College of Medicine and Health Sciences, An-Najah National University, Nablus, 44839 Palestine

**Keywords:** Hemodialysis, Treatment adherence, Perception, Clinical outcomes, Palestine

## Abstract

**Background:**

Adherence to diet recommendations, fluid restriction, prescribed medications, and attendance at hemodialysis (HD) sessions are essential for optimal and effective treatment of patients with end-stage renal disease. No data regarding this issue are available from Palestine. Therefore, this study was carried out to assess adherence to diet, fluid restriction, medications, and HD sessions.

**Methods:**

A cross-sectional study of HD patients at An-Najah National University Hospital was carried out during summer, 2016. Self-reported adherence behavior was obtained using a valid and reliable questionnaire (End-Stage Renal Disease Adherence Questionnaire: ESRD-AQ). Predialytic serum levels of potassium and phosphate were obtained as clinical indicator of diet and medication adherence respectively. In addition, interdialytic body weight (IDW) was also obtained from medical records and analyzed in relation to reported adherence of fluid restriction.

**Results:**

A total of 220 patients answered all questions pertaining to ESRD-AQ. The mean age ± standard deviation of participants was 56.82 ± 14.51 years. Dietary adherence was observed in 24% while that of fluid restriction adherence was observed in 31% of studied patients. Reported adherence to HD sessions was 52% while that for medications was 81%. Overall, 122 (55.5%) patients had good adherence, 89 (40.5%) had moderate adherence, and 9 (4.1%) had poor adherence behavior. Male patients had significantly higher overall adherence scores than females (*p* = 0.034). A significant correlation between reported diet adherence and serum pre-HD potassium level (*p* < 0.01) was observed. A significant correlation between reported fluid restriction adherence and IDW (*p* < 0.01) was also found. However, no significant correlation between reported adherence and pre-HD phosphate level. There was significant correlation between overall perception and overall adherence score (*p* < 0.001). Counselling of patients regarding importance of adherence modalities was lowest for “staying for the entire dialysis time”. Multivariate analysis indicated that elderly male patients who were city residents had higher odds of having higher adherence score.

**Conclusions:**

There was a good percentage of patients who had overall moderate or poor adherence. ESRD-AQ could be used to assess some aspects of HD adherence. Counselling and education of patients on HD are important to improve therapeutic outcome.

**Electronic supplementary material:**

The online version of this article (doi:10.1186/s12882-017-0598-2) contains supplementary material, which is available to authorized users.

## Background

Chronic kidney disease (CKD) is a progressive irreversible structural damage and/ or kidney function [1]. There are five categories of CKD in which stage 5 (End Stage Renal Disease (ESRD)) is the last and most serious stage [1]. In patients with ESRD, renal replacement therapy (RRT) such as long-term dialysis or kidney transplantation is needed for survival [2, 3]. Kidney transplantation is the best choice for management of patients with ESRD [4, 5]. However, the limited availability of organ donors made hemodialysis (HD) procedure as most efficient and practical method for management of patients with ESRD [6].

Patients on long-term HD are considered partially responsible for the success of their therapy by adherence to medication prescription, adherence to diet and fluid restrictions and complete adherence to HD sessions [7]. Failure of adherence in HD patients can lead to increase morbidity, mortality, cost, and burden on healthcare system [8–12]. Patients undergoing HD are required to maintain their potassium and phosphate serum level within a safe range to avoid serious complications such as fatal arrhythmia and osteodystrophy [13]. Furthermore, they are also required to maintain a limited amount of fluid intake to avoid edema and cardiovascular complications [13]. Several reports on adherence among HD patients have been published [11, 14–17]. Many other published studies reported lack of diet and medication adherence among patients undergoing HD [17–21]. Despite the importance of adherence in general population of patients and in HD patients in particular, there are very limited number of studies which discussed this topic at the local and regional level in Arab countries [22–24]. In Palestine, there are few studies on medication adherence in general and none about adherence in HD patients [25–33].

In Palestine, the majority patients with ESRD are treated as chronic HD patients in hospitals run by the Palestinian government which suffers from continuous financial problems and limited economic resources [34, 35]. It has been reported that risk factors for non-adherence includes health care system–related factors which have been neglected by most authors who assessed the subject [7, 36]. Since the healthcare system in Palestine and in the Arab region is different from that in the USA, Europe and in other developed countries, this necessitates the assessment of adherence among HD patients in Palestine and implementing the necessary changes and measures to overcome such a problem. Few years ago, the Palestinian Ministry of Health made an agreement with An-Najah National University Hospital in Nablus to carry out HD procedure for patients with governmental insurance in Nablus district. Currently, An-Najah National University Hospital provides nephrology services for approximately 220 patients with ESRD living in Nablus district. The hospital is equipped with up-to-date technology and has specialized and well – trained staff.

Assessing adherence among HD patients will allow healthcare providers to implement interventional methods to minimize health and economic consequences of non-adherence. The aim of this study was to assess extent of adherence among HD patients to different treatment modalities. The study was carried on patients attending HD center at An-Najah National University Hospital. In specific, the current study will assess adherence to fluid restriction, adherence to diet recommendations, adherence to medications, and adherence to HD schedules.

## Methods

This study was conducted in the main HD center in northern west-bank located at An-Najah National University Hospital in Nablus. The center offers HD services to a total population of more than 100,000 people living in Nablus district. The study included all patients ≥18 years of age who were conscious, have been on HD for at least 6 months, receive dialysis at least twice weekly with a minimum of 3 h per session, and his/her medical file contained all needed biochemical, clinical, and demographic information needed for this study. The study was cross-sectional and was carried out during the summer of 2016. For this study, all patients attending the HD unit were approached and asked to participate in the study. To achieve this, three of the co-authors spent 6 consecutive weeks at the dialysis center for the purpose of data collection. The three co-authors were senior medical students who were trained on the tool used to assess adherence. One co-author did the interviews and two co-authors collected information pertaining to biochemical and clinical information from medical files of interviewed patients. Patients included in the study were asked to give an informed consent based on the Institutional Review Board (IRB) approval of the study.

In this study, the ESRD-AQ (End Stage Renal Disease – Adherence Questionnaire) was used as a tool to assess: (1) degree of adherence, (2) perception, and (3) counselling of patients toward HD treatment modalities. The ESRD-AQ is a reliable and valid instrument used to assess adherence among HD patients [37]. The questionnaire consists of 46 items that were distributed into five sections: the first section contained general and history related information while the remaining four sections measures adherence to HD sessions, adherence to medications, adherence to fluid restriction, and adherence to diet recommendations. Questions number 14, 17, 18, 26, 31, and 46 were used to calculate the adherence behavior subscale. These questions were scored and response of patients to these questions was summed to calculate the adherence behavior subscale. According to ESRD-AQ, higher scores represents higher adherence to the measured behavior.

Questions number 11, 12, 22, 23, 32, 33, 41, and 42 were used to assess and describe the attitude/perception subscale. These questions were non-scored but the answers range from very high (given number 1) to very low (given number 5) with the possibility of presenting answers in a numerical way. In adherence subscale, higher scores indicate better adherence. For the clinical assessment of adherence, pre-dialytic serum potassium and phosphate levels were used along with interdialytic body weight (IDW) as clinical indicators for adherence to diet, adherence to medications, and adherence to fluid restriction respectively.

For the purpose of this study, the ESRD-AQ scale was translated into Arabic by two colleagues who are USA graduates with Arabic being their native language. Then the translated scale was back translated to English and compared by the authors with original copy to make sure that none of the questions lost any of its intended meaning. The Arabic - translated ESRD-AQ was not validated although the original English version of ESRD-AQ was validated. The Arabic – translated version of the ESRD-AQ is provided in Additional file 1.

Measured clinical outcomes included biochemical markers of pre-HD serum phosphorus and potassium. Mean ± standard deviation of pre-HD serum potassium level was used as a biochemical marker for adherence to diet recommendation while mean value for pre-HD serum phosphate level was used as a biochemical marker for medication (phosphate binders, i.e. calcium carbonate) adherence. For all biochemical markers, average of the last three measurements carried out in the past month was used. Finally, the IDW was calculated by subtracting the post-HD weight from the pre-HD weight which represents fluid consumption from one dialysis session to the next. The mean of three consecutive IDW was used as a biochemical marker for fluid restriction adherence.

## Statistical analysis

Statistical Package for Social Sciences version 16 (SPSS Inc., Chicago, IL, USA) was used for data processing. Mean ± standard deviation and/ or median (first quartile – third quartile) were used to describe data after checking for normality of data which was tested using Kolmogorov test. Correlations between variables were tested using Spearman correlation and both significance (*p*) and correlation coefficient (r) were presented whenever appropriate. Comparison of adherence between males and females was carried out using Mann – Whitney U test. Predictors of adherence were obtained by using multiple linear regression. The independent factors used in the model were gender, marital status, residency, living status, type of transportation to HD centre, and the duration of illness (ESRD). These were all demographic and clinical variables obtained directly from studied patients. The significance level was predetermined at p level < of 0.05 for all tests.

## Results

### General information

A total of 223 met the inclusion criteria and were recruited for the study. However three patients died during the study period and were excluded from analysis. Therefore, a total of 220 patients were recruited and interviewed. The total number of patients recruited represented 98.65% of eligible patients at the time of study. Studied patients had a mean age of 56.82 ± 14.51 years with a range of 18–85 years. More than two thirds of studied patients (159, 72.3%) were under 65 years of age. The majority of studied sample were males (128, 58.2%), married (189, 85.9%), and city residents (102, 46.4%). Approximately one third (74; 33.64%) of studied patients were diabetic hypertensive, 122 (55.46%) had hypertension and 87 (39.55%) patients had diabetes mellitus (DM). A total of 136 (61.82%) of studied patients had either DM, or hypertension or both. A total of 85 (38.6%) patients had no chronic diseases as a known cause of ESRD. Twenty patients (9.1%) had previous kidney transplant. Approximately two thirds (149. 67.7%) of the patients used public transportation to reach the dialysis center and the majority (102, 46.4%) attended the dialysis sessions alone without any family company. The majority of studied patients (203, 92.27%) described their dialysis schedule as convenient. The mean number of months of dialysis as reported by studied patients was 48.16 ± 44.41 with a range of 12–408 months. Table 1 shows selected socio-demographic and clinical characteristics of the study sample.

**Table 1:** Selected socio-demographic and clinical characteristics of the study sample

Variable	Mean ± SD or number of patients (%)
Age (mean ± SD)	56.82 ± 14.51
Gender	
Male Female	128 (58.2) 92 (41.8)
Marital status	
Married Single/widowed/divorced	189 (85.9) 31 (14.1)
Education level	
≤ High school ≥ College/university	208 (94.5) 12 (5.5)
Residency	
City Suburbs (Palestinian refugee camps or village)	102 (46.4) 118 (53.6)
Duration of dialysis (mean ± SD) months	48.2 ± 44.4
Diabetes mellitus	
Yes No	87 (39.6) 133 (60.4)
Hypertension	
Yes No	122 (55.5) 98 (44.5)
Diabetes mellitus and hypertension	
Yes No	74 (33.6) 146 (66.4)
Kidney transplant	
Yes No	20 (9.1) 200 (91.9)
How he/she reached the center	
Public transportation Private/ others (ambulance)	152 (69.1) 68 (30.9)
Accompanied by family to HD center	
Yes No (Alone)	118 (53.6) 102 (46.4)

*Abbreviations*: *SD* Slandered deviation, *HD*, hemodialysis

### Specific and overall adherence

Adherence to the four treatment modalities was assessed. Adherence to HD sessions was the highest with an average score of 296.36 ± 26.78 out of a maximum score of 300. Adherence to medications was also assessed and the mean score was 184.32 ± 37.83 out of a maximum score of 200. Mean adherence score for fluid restriction was 140.68 ± 53.78 out of a maximum score of 200. Mean adherence score to diet recommendations was 134.55 ± 52.01 out of a maximum score of 200 (Table 2).

**Table 2:** Mean (SD) adherence score for various treatment modalities

Item # in ESRD-AQ	Adherence	Range of score	Mean score (SD)
14	HD - attendance	100–300	296.36 (26.78)
17	Episode of shortening HD	0–200	149.32 (68.83)
18	Duration of shortening HD if shortened	0–100	80.11 (27.54)
26	Adherence to medication	0–200	184.32 (37.83)
31	Adherence to fluid restriction	0–200	140.68 (53.78)
46	Adherence to dietary restriction	0–200	134.55 (52.01)

*Abbreviations*: *SD* Slandered deviation, *HD* hemodialysis, *ESRD-AQ* End-Stage Renal Disease Adherence Questionnaire

Overall adherence behavior of each patient was assessed by summing the scores of questions 14, 17, 18, 26, 31, and 46. A total of 122 (55.5%) patients had good overall adherence behavior, 89 (40.5%) had moderate adherence and nine patients (4.1%) had poor adherence (Table 3). There was no statistically significant correlation between adherence behavior and age (Spearman correlation: *p* = 0.27, *r* = 0.074) or number of months since starting (Spearman correlation: *p* = 0.98, *r* = −0.002). However, male patients had significantly higher adherence behavior score compared to female patients (male = 1003.13 ± 144.18; female = 960.60 ± 154.74; *p* = 0.034).

**Table 3:** Overall adherence and End-Stage Renal Disease Adherence Questionnaire (ESRD-AQ) score

Adherence category	Total Score	Frequency	Percent
Poor	< 700	9	4.1
Moderate	700–999	89	40.5
Good	1000–1200	122	55.5

### Clinical outcomes

The mean ± SD for pre-HD serum potassium of studied patients was 1.27 ± 0.19 meq/l while the median (Q1–Q3) was 1.28 (1.13–1.41) meq/l. There was a significant negative correlation between pre-dialytic serum potassium level and diet adherence score (Spearman correlation: *p* < 0.001; *r* = − 0.28). The mean IDW was 3.10 ± 1.63 Kg while the median (Q1–Q3) was 3.0 (2–4) Kg. There was a significant negative correlation between IDW and adherence to fluid restriction (Spearman correlation: *p* < 0.001, *r* = − 0.423). The mean (SD) pre-dialytic phosphate level was 1.56 ± 0.54 while the median (Q1–Q3) was 1.51 (1.28–1.76) meq/L. The levels of serum phosphate are within high normal level indicative of denutrition. There was no significant correlation between medication adherence and pre-dialytic phosphorous level (Spearman correlation: *p* = 0.29) (Tables 4 and 5).

**Table 4:** Correlations between reported adherence and clinical outcomes

Variables^a^	Pre-dialysis Phosphate level	Pre-dialysis Potassium level	IDW	Diet adherence score	Fluid restriction adherence score
Pre-dialysis Potassium level	.331				
	**<0.001** ^b^				
IDW	.137	.210			
	**0.042** ^b^	**0.002** ^b^			
Diet adherence score	−.108	−.281	−.270		
	.110	**<0.001** ^b^	**<0.001** ^b^		
Fluid restriction adherence score	−.081	−.128	−.423	.408	
	.229	.058	**<0.001** ^b^	**<0.001** ^b^	
Medication adherence score	.071	.042	−.143	.004	.043
	.292	.540	**0.035** ^b^	.954	.526

^a^For each pair of correlation, the number in the top cell represents the Spearman correlation value while the number in the bottom cell represents the significance level

^b^The *p*-values are bold where they are less than the significance level cut-off of 0.05

**Table 5:** Summary of correlation results

Correlation	Result
Potassium and adherence to diet recommendation	Significant
Potassium and adherence to fluid restriction	Significant
Potassium and medication adherence	Not significant
IDW and adherence to diet recommendation	Significant
IDW and adherence to fluid restriction	Significant
IDW and medication adherence	Not Significant
Phosphate and adherence to diet recommendation	Not Significant
Phosphate and adherence to fluid restriction	Not Significant
Phosphate and medication adherence	Not Significant

A significant correlation between pre-dialytic serum potassium level and total adherence behavior score (*p* = 0.028, *r* = − 0.15) was found. Similarly, a significant correlation between IDW and adherence behavior score (*p* < 0.001, *r* = 0.37) was found. However, no significant correlation between phosphate and total adherence behavior score was found. No significant correlation was found between total number of dialysis hours per week in one hand and any of the treatment modalities on the other hand.

### Perception of patients toward various HD treatment modalities

We assessed the perception of studied patients toward various HD treatment modalities (Table 6). Perception toward HD sessions had the highest score with 96.4% of studied patients believe that it is highly/very important to follow the dialysis schedule. Perception toward diet restriction was the lowest with 77.7% of studied patients believe that it is highly/ very important to watch the type of food taken daily. The perception of importance toward medication adherence and fluid restriction were comparatively fair with 85.5% and 88.6% of the studied patients believe that it is highly/very important to adhere to medications and restrict fluid intake respectively.

**Table 6:** Perception on importance of adherence to various treatment modalities

Item # in ESRD-AQ	Perception on importance	Highly/Very important *n* (%)	Moderately important *n* (%)	Little/Not important *n* (%)
11	How important do you think it is to follow your dialysis schedule?	212 (96.3)	6 (2.7)	2 (1)
22	How important do you think it is to take your medicines as scheduled?	188 (85.5)	22 (10)	10 (4.5)
32	How important do you think it is to limit your fluid intake?	195 (88.6)	17 (7.7)	8 (3.7)
41	How important do you think it is important for you to watch your diet daily?	171 (77.7)	29 (13.2)	20 (9.1)

*Abbreviation: ESRD-AQ* End-Stage Renal Disease Adherence Questionnaire

The sum of perception scores obtained by summing questions 11, 22, 32 and 41 yielded a median of 6 (Q1–Q3 = 5–8) and a mean of a 6.86 ± 2.39. Of course the lower the total score of perception, the better attitude the patients had toward HD treatment modalities. Spearman correlation between total perception score and total adherence score yielded a significant negative correlation (*p* < 0.001, *r* = − 0.446) suggesting that better perception and attitude yields better adherence score.

## Counselling

**Fig. 1:**
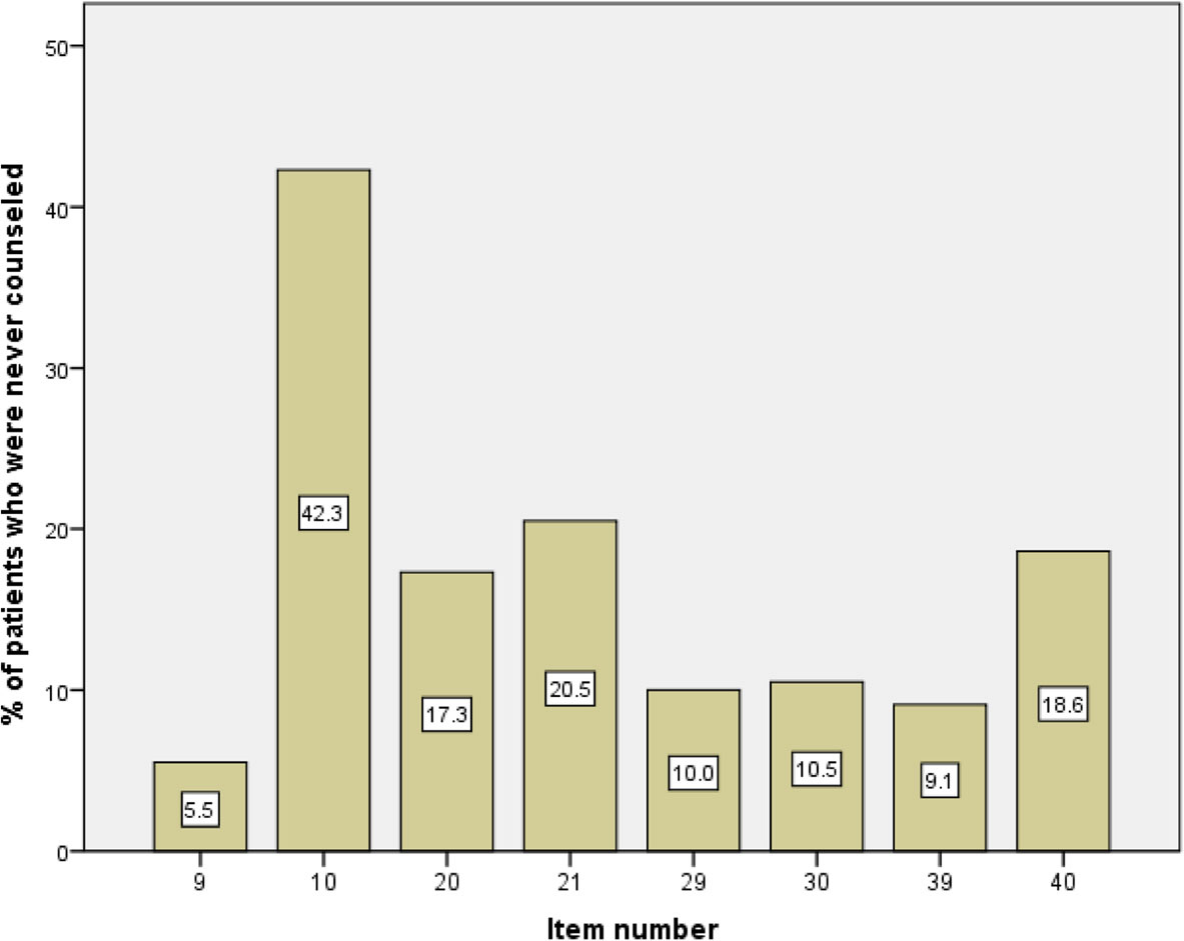
Percentage of patients who were never counseled regarding different treatment modalities. Item # 9: When was the last time a medical professional (your doctor, nurse, dietician, or other medical staff) talked to you about the importance of not missing your dialysis treatment? Item # 10: How often does a medical professional (your doctor, nurse, dietician, or other medical staff) talk to you about the importance of staying for the entire dialysis time during your dialysis treatment? Item # 20: When was the last time a medical professional (your doctor, nurse, dietician or other medical staff) spoke to you about your medicines? Item # 21: How often does a medical professional (your doctor, nurse, dietician or other medical staff) talk to you about the importance of taking medicines as ordered? Item # 39: When was last time a medical professional (your doctor, nurse, dietician, or other medical staff) talked to you about your diet? Item # 40: How often does a medical professional (your doctor, nurse, dietician or other medical staff) talk to you about the importance of following a proper diet?

Eight items in the ESRD-AQ discuss the counselling received by patients for various treatment modalities. For each treatment modality, the patients were asked two questions pertaining to counseling. The most negative answer was “never”. Approximately 42% of studied patients reported that they had never been talked to by a healthcare provider about “the importance of staying for the entire dialysis time during dialysis treatment”. Regarding medication counselling, approximately 21% of studied patients reported that they had never been talked to by a healthcare provider regarding their medications. Similarly, 19% reported that they had never been talked to by a healthcare provider regarding the importance of following a diet restriction. The counselling for other treatment modalities is shown in Fig. 1.

### Predictors of adherence

Multivariate analysis indicated significant association between a number of independent variables and adherence score in comparison to a reference category for categorical variables or with one unit increase for a continuous variable. Age, gender, and residence were positively associated with adherence scores. In specific, elderly male patients who lives in the city have higher odds of having higher overall adherence score (Table 7).

**Table 7:** Multiple linear regression analysis of association between factors and adherence score

Variables	Unstandardised coefficients (B)	Standardised coefficients (Beta)	*P* value^a^	95% CI for B
Age				
Continuous (1-year units)	1.78	0.17	**0.021**	0.28–3.29
Gender				
Male Female	44.88 Reference	0.15	**0.038**	2.58–87.17
Marital status				
Married Widowed /divorced	26.11 Reference	−0.06	0.431	−91.29–39.07
Residency				
City Palestinian refugee camps or village	51.32 Reference	0.17	**0.015**	9.89–92.75
Duration of dialysis				
Continuous (1-month units)	0.14	0.04	0.615	−0.41–0.69
Dialysis centre transportation				
Public transportation Private/ ambulance	18.39 Reference	−0.06	0.402	−61.59–24.80
Living statues				
With family Alone	−0.13 Reference	0.00	0.995	−43.53–43.27

*CI* confidence interval

^a^The *p*-values are bold where they are less than the significance level cut-off of 0.05.

## Discussion

In the current study, adherence behaviors among patients on maintenance HD were investigated and analyzed. The findings of our study showed that adherence to HD treatment modalities was less than optimum with approximately 45% of studied patients had an overall moderate or poor adherence. A significant correlation between reported diet adherence and pre-HD serum potassium level is suggestive of the validity of reported adherence scores. Similar significant correlation existed between reported fluid restriction adherence and IDW. Our study showed that perception of importance of adherence was significantly correlated with reported adherence suggesting that counselling of patients on HD regarding their treatment modalities is important to improve therapeutic outcome. Our study indicated that male elderly patients who live in the city have higher odds of having higher adherence.

From a health point of view, the majority of patients (55%) were hypertensive and 39% were diabetic patients. A study carried out in Saudi Arabia found that most of HD patients were diagnosed with hypertension and more than one third were diagnosed with DM. A study carried out in India found that 81% of HD patients were hypertensive and 24% were diabetics. Differences in co-morbid diseases among patients with HD in various studies are probably due to differences in ethnic and cultural environment of the study sample. It should be emphasized that patients in our study were homogenous; all patients were Palestinians from one district which is in contrast to studied sample in other studies where patients had different ethnic background.

In our study, there was a significant correlation between reported adherence to fluids or dietary recommendation and clinically determined adherence. This suggests that reported fluid or diet adherence calculated by ESRD-AQ is trustful given the significant correlation with clinically determined adherence parameters. However, no significant correlation between pre-dialytic serum phosphate with any reported adherence behavior was found. This suggests that ESRD-AQ might not be a good tool to assess medication adherence or that pre-dialytic phosphate serum level was not a suitable clinical indicator for reported medication adherence among studied patients. Actually, medications for HD patients are dispensed in a complex manner that might make their answers to medication adherence inaccurate. Studied patients receive HD at An-Najah National University Hospital and receive their medications from governmental pharmacies since they are covered by governmental insurance. Furthermore, patients sometimes fail to adhere to medications simply because medications are not always available at the hospital. So, despite the fact that a patient might perceive himself/herself as fully adherent, he/she might not be taking their medications because the medications were not dispensed due to lack of medications.

Results obtained from various studies on adherence among HD patients vary widely which made comparison among different studies a difficult task. Several studies were published from different world regions regarding prevalence of treatment adherence among HD patients. A study in Malaysia found that rates of adherence to fluid, dietary, medication, and dialysis were 27.7, 66.5, 24.5, and 91.0%, respectively [38]. A study in Makah city found that the prevalence of adherence to fluid restrictions recommendations, dietary, and medication prescription among HD patients were 87.78, 88.37, and 87.99%, respectively [39]. Nearly half of patients reported in Makah study were adherent to dialysis sessions (55.96%) [39]. A study in China in HD patients found that fluid and dietary adherence were seen in 40.3% and 35.5% respectively [40].

In our study, it was noticed that adherence to HD attendance, mainly shortening of HD sessions, was relatively low. In this regard, it seems that patients have been poorly counseled regarding completing HD sessions since almost 42% of patients reported that they have never been counseled in this regard. It seems that patients’ education and counselling are important in formulating patients’ general perception toward various treatment aspects which in turn can significantly affect patients’ adherence. In this study, counselling regarding medications and diet restriction were not high which in turn created relatively lower perception toward various treatment aspects among studied patients.

Our study showed that older patients and male patients have higher odds of being adherent. Some studies showed similar findings regarding age but found no effect of gender on level of adherence [22, 41]. In contrast to the finding presented in our study, marital status was found to be a detrimental factor for adherence [42]. In our study, the level of education was not entered in the regression model since the vast majority of the study sample had the same level of school education and a small minority had college or university education. Other studies have found a positive role of education on level of adherence [7].

Our study had a few limitations that are inherent to the nature of the study design and the self-reported approach of the tool used in the study. Reports indicated that there is a disagreement between self-reported adherence and actual/observed adherence [43, 44]. A second limitation of our study is the lack of validated Arabic-translated version of the ESRD-AQ scale. A third limitation is the absence of a universally accepted cutoff value for each biological marker to be a valid point for identification of adherent versus non-adherent patients. Therefore, the validity of these biological markers to assess adherence in ESRD patients might be questionable although these markers may be more effective or reliable measures of clinical outcomes but not necessarily be adequate for measuring non-adherence. Finally, our study was a single center study and we hope that future research will be designed to include all HD patients in Palestine.

## Conclusions

Our study showed that adherence to HD treatment modalities is less than optimum. Approximately 45% of studies patients had overall moderate or poor adherence. A significant correlation between reported diet adherence and pre-HD serum potassium level is suggestive of the validity of reported adherence scores. Similar significant correlation existed between reported fluid restriction adherence and IDW. Our study showed that perception of importance of adherence is significantly correlated with reported adherence suggesting that counselling and education of patients on HD regarding their treatment modalities is important to improve therapeutic outcome. Finally, male older patients who were city residents had higher odds of being more adherent.

## Additional file

Additional file 1: Arabic version of ESRD-AQ. This file is the Arabic translated version of ESRD-AQ scale used in the current study. The translation process is explained in the methodology section. (DOC 90 kb)

## Abbreviations

CKD: Chronic kidney disease
DM: Diabetes mellitus
ESRD: End Stage Renal Disease
ESRD-AQ: End-Stage Renal Disease Adherence Questionnaire
IDW: Inter-dialytic body weight
IRB: Institutional Review Board
RRT: Renal replacement therapy
SPSS: Statistical Package for Social Sciences
