# Supplementary material for: Treatment adherence and perception in patients on maintenance hemodialysis: a cross – sectional study from Palestine
Source: BMC Nephrol. 2017 May 30;18:178. doi: 10.1186/s12882-017-0598-2 (PMC5450383; doi:10.1186/s12882-017-0598-2)
Supplement: Additional file 1: — Arabic version of ESRD-AQ. This file is the Arabic translated version of ESRD-AQ scale used in the current study. The translation process is explained in the methodology section. (DOC 90 kb) [file 12882_2017_598_MOESM1_ESM.doc]

**­ Additional file 1: Arabic version of ESRD-AQ.** This file is the Arabic translated version of ESRD-AQ scale used in the current study. The translation process is explained in the methodology section.

**استبيان لالتزام مرضى الكلى (ESRD)**

**الاسم:________________ العمر:____ الجنس:_____
الحالة الاجتماعية:_________ المستوى التعليمي:_________
سبب الفشل الكلوي:_________
الوزن الزائد:____________ أمراض مزمنة:________________
زراعة كلى سابقة؟_________ بوتاسيوم قبل\بعد:________\_______
الإقامة:_________ رقم الهاتف\النقال:________________**

**القسم الأول: معلومات عامة**

1. **متى بدأت غسيل الكلى؟** بدأ \ استكمل في شهر_____\سنة_____
2. **هل سبق وأن عملت غسيل عن طريق البطن (Peritoneal dialysis)؟ 1-** لا **2-** نعم
    (شهر______\سنة______ إلى شهر _____\سنة______)
3. **هل سبق وزرعت كلية؟ 1-** لا **2-** نعم (المرة الأولى من _____\_____ حتى _____\______)
    (المرة الثانية من _____\_____ حتى _____\_____) - **إن كان أكثر من 2 اكتب آخر 2**
4. **كيف تصل إلى مركز غسيل الكلى؟**  **1-** مواصلة خاصّة **2-** حافلات **3-** تاكسي **4-** سيارات نقل طبية (اسعاف مثلاً) **5-** غير ذلك______
5. **من يرافقك إلى مركز غسيل الكلى؟
    1-** لوحدي (لا أحد) **2-** الوالد\ة **3-** الزوج\ة **4-** الابن\ة **5-** صديق\ة **6-** غير ذلك______

**القسم الثاني: معلومات عن غسيل الكلى**

1. **كم يوم بالأسبوع تخضع لغسيل الكلى؟** **أ-** 2 **ب-** 3 **ج-** 4 **د-** أكثر من 4 أيام **هـ -** أكثر من 5 أيام
2. **كم عدد ساعات غسيل الكلى؟
   أ-** أقل من 3 **ب-** 3 **ج-** 3 وربع **د-** 3 ونص **هـ -** 3 و45 د. **و-** 4 **ز-** أكثر من 4 **ح-** غيرذلك___
3. **هل موعد الغسيل مناسب بالنسبة لك؟** **1-** نعم **2-** لا؛ لإني أضطر للقدوم باكراً جداً **3-** لا؛ لإني أضطر متأخراً جداً
   **4-** لا؛ لإنه يتضارب مع عملي **5-** لا؛ لإنه يتعارض مع وقت وجبتي (أجوع خلال فترة الغسيل)
    **6-** لا؛ لإنه يتعارض مع وقت تناول أدويتي\أنسولين **7-** غير ذلك__________
4. **متى كانت آخر مرّة تحدّث معك طبيبك\ممرضك\عامل صحّي معك عن أهمية إلتزامك بالغسيل وعدم تفويت أحدها؟
   1**- هذا الأسبوع **2-** الأسبوع الماضي **3-** قبل شهر **4-** قبل أكثر من شهر **5-** أبداً لم يتحدّث **6-**غيرذلك__
5. **بالعادة كم مرة يتحدّث معك طبيبك\ممرضك\عامل صحّي معك عن أهمية إلتزامك بوقت الغسيل (خلال فترة الغسيل)؟**
   **1-** كل مرة أخضع فيها للغسل **2-** كل أسبوع **3-** كل شهر **4-** كل 2-3 أشهر  **5**- كل 4-6 شهور
   **6-** عندما تظهر نتائج غير طبيعية بالفحوصات (دم أو غيره) **7-** نادراً **8-** بشكل غير منتظم
   **9-** أبداً لم يتحدّث **10-** غيرذلك____
6. **برأيك، ما مدى أهمية غسيل الكلى (قيّم من 1-5)؟** **أ-** مهم جداً- 5 **ب-** مهم-4 **ج-** متوسط الأهمية–3 **د-** قليل الأهميّة–2 **هـ -** ليس مهماً-1
7. **برأيك، لم من المهم اتباع جدول غسيل الكلى الخاصة بك؟**
   **1-** لأني أفهم تماماً أنّ حالة الكلى خاصتي تتطلب غسيل الكلى كما هو مقرر
   **2-** لأنّ إتّباع جدول الغسيل مهمّ للحفاظ صحّتي **3-** لأن عامل الصحّة (طبيب\ممرض\أخصّائي تغذية) قال لي ذلك
   **4-** لأنّني سبق ومرضت بعد أن غبت عن غسيل الكلى
   **5-** لأنّني سبق ودخلت (نمت) بالمستشفى بعد أن غبت عن غسيل الكلى
   **6-** لا أعتقد أن الالتزام بجدول الغسيل مهم. **7-** غيرذلك_____
8. **ما مدى الصعوبة التي واجهتها لتستمرّ بوقت الغسيل بأكلمه (كما طلب الطبيب)؟ (قيّم من 1-5)**
   **أ-** لا صعوبة-1 **ب-** صعوبة قليلة-2 **ج -** متوسطة–3 **د-** صعوبة كبيرة – 4 **هـ -** صعوبة متناهية – 5
9. **خلال الشهر الماضي، كم مرّة غبت عن موعد الغسيل كليّاً؟**
    **أ-** لم أغب **ب-** مرة **ج-** مرتين **د-** ثلاث مرّات **هـ -** 4 مرّات أو أكثر
10. **ماذا كان السبب الرئيسي وراء غيابك عن الغسيل بالشهر الماضي؟**
    **1-** لم أغب  **2-** مشاكل بالمواصلات  **3-** انشغلت بشيء آخر (التوضيح_______________)
    **4**- تخثّر في وصلة الغسيل (AV shunt\Catheter)  **5-** موعد مع طبيب (جراحة\عيادة\عملية)
     **6-** كنت مضطراً للذهاب إلى الطوارئ  **7-** لم أرغب في الذهاب أو لم أستطع الذهاب (التوضيح)_______________
11. **(في حال كانت إجابة السؤال السابق لم أرغب أو لم أستطع) لِمَ غبت غسيل الكلى السابق؟
    1-** لأنّ الغسيل يوتّرني  **2-** لأنّي كنت أتقيّأ أو أعاني من إسهال  **3-** لأنّي كنت أعاني من مغص
    **4-** لأني عادة أجوع خلال الغسيل  **5-** لأنّي كنت مرهقاً جسديّا ً
    **6-** لأنّي كنت مريضاً لأسباب أخرى ____________  **7-** غير ذلك_______________
12. **خلال الشهر الماضي، كم مرّة تم تقصير مدّة (وقت) غسيل الكلى؟
     أ-** لم يتم ذلك  **ب-** 1  **ج-** 2  **د-** 3  **هـ -** 4-5  **و-** غير ذلك_____
13. **خلال الشهر الماضي، في حال كان قد تم تقصير وقت الغسيل، كم كان معدّل الدقائق التي تم تقصيرها؟**
    **أ-** لم يتم ذلك  **ب-** 10 دقائق أو أقل  **ج-** 11-20 دقيقة  **د-** 21-30 دقيقة  **هـ -** أكثر من 31 دقيقة  **و-** غير ذلك_______
14. **ماذا كان السبب الرئيسي وراء تقصير وقت الغسيل؟
    1-** لم يتم ذلك  **2-** مغص، تشنجات (cramping)  **3-** لاستخدام الحمّام
    **4-** ضجر،هياج (restlessness)  **5-** انخفاض في ضغط الدم
     **6-** تخثّر في وصلة الغسيل (AV shunt/Catheter)  **7-** موعد مع الطبيب (جراحة\عيادة\عملية) **8-** عمل شخصي أو حالة طارئة  **9-** عمل جدولي (مجدول)  **10-** مشكلة مواصلات
    **11-** قرار من الطاقم المسؤول (التوضيح_________)  **12-** لم أرغب في البقاء
    **13-** غير ذلك _______________

**القسم الثالث: الأدوية**

1. **متى كانت آخر مرّة تحدّث معك طبيبك\ممرضك\عامل صحّي معك عن أدويتك؟
   1-** هذا الأسبوع  **2-** الأسبوع الماضي  **3-** قبل شهر  **4-** قبل أكثر من شهر  **5-** عندما بدأت غسيل الكلى  **6-** أبداً **7-** غير ذلك________
2. **كم بالعادة يتحدّث معك طبيبك\ممرضك\عامل صحّي معك عن أهمية إلتزامك بأخذ الأدوية وعدم تفويت أحدها؟
   1-** في كل غسيل  **2-** كل أسبوع **3-** كل شهر  **4-** كل شهرين إلى 3 شهور **5-** كل 4 - 6 شهور   **6-** عندما تظهر نتائج غير طبيعية بالفحوصات (دم أو غيره)  **7-** نادراً  **8-** بشكل غير منتظم
   **9-** أبداً لم يتحدّث  **10-** غير ذلك____
3. **برأيك، ما مدى أهمية تناولك للأدوية (قيّم من 1-5)؟
   أ-** مهم جداً- 5  **ب-** مهم-4  **ج-** متوسط الأهمية–3  **د-** قليل الأهميّة–2  **هـ -** ليس مهماً-1
4. **برأيك، لم من المهم الالتزام بتناول الأدوية التي تم وصفها من قبل الطبيب؟**
   **1-** لأني أفهم تماماً أنّ حالة الكلى خاصتي تتطلب الأدوية كما هو مقرر
   **2-** لأنّ الالتزام بتناول الأدوية مهمّ للحفاظ صحّة جسدي
   **3-** لأن عامل الصحّة (طبيب، ممرض، أو اختصاصي تغذية) قال لي ذلك
   **4-** لأنّني سبق ومرضت بعد أن توقفت عن تناول الأدوية
   **5-** لأنّني سبق ودخلت (نمت) بالمستشفى بعد أن توقفت عن تناول الأدوية
   **6-** لا أعتقد أن الالتزام بالأدوية مهم.  **7-** غير ذلك___________
5. **هل تواجه صعوبة في تناول أدويتك؟** **1-** لا  **2–** نعم
6. **ما مدى الصعوبة التي واجهتها لتلتزم بتناول الأدوية (كما طلب الطبيب)؟ (قيّم من 1-5)**
   **أ-** لا صعوبة-1 **ب-** صعوبة قليلة-2 **ج-** متوسطة–3 **د-** صعوبة كبيرة–4 **هـ -** صعوبة متناهية–5
7. **خلال الاسبوع الماضي , كم مرة لم تتذكر "نسيت" أخذ الدواء الموصوف لك؟
   1-** ولا مرة **2-** نادر جدا **3-** تقريبا نصف المرات **4-** أغلب الأوقات **5-** كل الأوقات
8. **ما هو السبب الرئيس وراء عدم أخذك الدواء الموصوف خلال الاسبوع الماضي؟
   1-** ولا مرة (لم ينسى أخذ الدواء) **2-** نسيان أخذ الدواء **3-** نسيان طلب الدواء
   **4-** تكلفة الدواء **5-** الأمر مزعج **6-** كنت في المستشفى
   **7-** الآثار الجانبية للأدوية (اذهب ل سؤال 28) **8-** أسباب أخرى........................
9. ***هذا السؤال فقط اذا كان جواب 27 هو الاثار الجانبية للادوية*** – **ما هي الاثار الجانبية للادوية التي تشكو منها (الرجاء اختيار اكثر اجابة "واحدة" تتناسب مع المريض):
   1-** فقدان الشهية **2-** (غثيان , قيء , اسهال , امساك ) **3-** الم معدة **4-** دوخة (دوار)
   **5-** وجع رأس **6-** حكة أو مشاكل جلدية **7-** أعراض أخرى..........................

**القسم الرابع: السوائل**

1. **متى كانت آخر مرّة تحدّث معك طبيبك\ممرضك\عامل صحّي معك عن حمية السوائل؟
   1-** هذا الأسبوع **2-** الأسبوع الماضي **3-** الشهر الماضي **4-** قبل أكثر من شهر
   **5-** عندما بدأت بغسيل الكلى **6-** ولا مرة **7-** أوقات أخرى .............
2. **كم مرّة يتحدّث معك طبيبك\ممرضك\أخصّائي التغذية\عامل صحّي معك عن أهميّة حمية السوائل؟** **1-** كل جلسة غسيل **2-** كل أسبوع **3-** كل شهر **4-** كل 2-3 أشهر  **5**- كل 4-6 شهور
    **6-** عندما تظهر نتائج غير طبيعية بالفحوصات (دم أو ضغط أو غيره) **7-** نادراً
   **8-** بشكل غير منتظم **9-** أبداً لم يتحدّث **10-** غير ذلك____
3. **خلال الأسبوع الماضي كم مرة اتبعت تعليمات القيود الخاصة بالسوائل ؟
   1-** كل الأوقات **2-** أغلب الأوقات **3-** تقريبا نصف الأوقات **4-** نادر جداً **5-** ولا مرّة.
4. **حسب اعتقادك، ما مدى أهمية الحد من تناول السوائل؟ (قيم من 1-5)
   أ-** مهم جدا جداً (5) **ب-** مهم جداً (4) **ج-** مهم (3) **د-** ليس مهم كثيرا (2) **هـ -** ليس مهم (1)
5. **لماذا تعتقد انه من المهم بالنسبة لك الحد من تناول السوائل ؟** (الرجاء اختار أفضل إجابة تتفق معك )
   **1-** لأني أتفهّم تماماً أنّ وضع كليتي يتطلب الحد من تناول السوائل
   **2-** لان الحد من تناول السوائل ضروري للبقاء على الجسم صحياَ
   **3-** لأنّ المختصّين في المجال الصحي أخبرني بالالتزام بذلك
   **4-** لأنه في السابق , مرضت بعد تناول كمية كبيرة من السوائل
   **5ـ** لأنّه في السابق دخلت المشفى بعد تناول كمية كبيرة من السوائل
   **6-** لا أعتقد أنّ الحد من تناول السوائل مهم جدا بالنسبة لي **7-** أسباب أخرى ...........................
6. **هل كان لديك أي صعوبة في الحد من تناول السوائل؟** لا // نعم
7. **كم** **واجهت من الصعوبة بخصوص حمية السوائل الموصى بها؟ ( قيم من 1-5)
   أ-** لم اواجه صعوبة (1) **ب-** صعوبة صغيرة (2) **ج-** صعوبة متوسطة (3)
   **د-** الكثير من الصعوبة (4) **هـ -** كنت غير قادر على الالتزام بالتوصيات على الإطلاق (5)
8. **إن كان هناك صعوبة بخصوص حمية السوائل الموصى بها**، **ما نوع هذه الصعوبة؟
   1-** لا يوجد صعوبة **2-** غير مهتم **3-** لم أكن أستطيع السيطرة على تناول السوائل
   **4-** لا أفهم كيف اتبع حمية السوائل الموصى بها  **5-** غير ذلك...........................
9. **خلال الأسبوع الماضي, كم مرة وزنت نفسك في البيت (خارج مركز الغسيل)؟
   1-** أكثر من ٣ مرات **2-** ٣ مرات **3-** مرتين **4-** مرة واحدة **5-** ولا مرة **6-** غير ذلك ــــــــــــ
10. **كم تعتقد أنّه من المهم أن توزن نفسك يومياً؟ (قيم من ١ إلى ٥)
    1-** مهم جداً **2-** كثير الأهمية **3-** متوسط الأهمية **4-** قليل الأهمية **5-** ليس مهماً

**القسم الخامس: الحمية الغذائية**

1. **متى كانت آخر مرّة تحدّث فيها طبيبك\ممرضك\عامل صحّي معك عن الحمية الغذائية؟
   1-** هذا الأسبوع **2-** خلال الأسبوع المنصرم **3-** قبل شهر **4-** قبل أكثر من شهر
   **5-** عندما بدأت أول جلسة غسيل **6-** أبداً **7-** أخرى ـــــــــــــــــــــــــــــ
2. **كل متى يتحدث معك طبيبك\ممرضك\عامل صحّي عن أهمية إتباع الحمية الغذائية؟
   1-** كل جلسة علاج **2-** كل أسبوع **3-** كل شهر **4-** كل شهرين إلى ثلاثة أشهر
   **5-** كل أربعة شهور إلى ستة شهور
   **6-** عندما تظهر نتائج غير طبيعية بالفحوصات (دم أو ضغط أو غيره)
   **7-** نادراً **8-** ليس بانتظام **9-** أبداً **10-** أخرى ــــــــــــــــــــــــ
3. **برأيك ما مدى أهميّة مراقبة أنواع الطعام الذي تتناوله يومياً؟ (قيم من ١ الى ٥)
   1-** مهم جداً **2-** كثير الأهمية **3-** متوسط الأهمية **4-** قليل الأهمية **5-** ليس مهماً
4. **لماذا تعتقد أنّه من المهم إتباع الحمية يومياً؟ (رجاءً اختار أفضل إجابة تنطبق عليك)
   1-** لأني أتفهّم تماماً أنّ وضع كليتي يتطلب إتّباع الحمية
   **2-** لأنّ إتباع الحمية مهم جداً للمحافظة على جسدي سليماً **3-** لأنّ طبيبي طلب مني ذلك **4-** لأنّي أشعر بالمرض عندما أتناول أنواع الطعام التي ليس من المفترض أن أتناولها
   **5ـ** لأنّه في السابق دخلت المشفى جرّاء تناولي أنواع طعام ليس من المفترض تناوله
   **6-** لا أعتقد أنّ إتباع الحمية مهم جدا بالنسبة لي **7-** أسباب أخرى .................................

Pre-Dialytic weight=
Post-Dialytic weight=
Potassium in past 3 months:
__________
__________
__________ AVG =_______
PO4- in past 3 months:
__________
__________
__________ AVG =_______

1. **هل واجهت صعوبات بخصوص إتبّاع الحمية الموصى بها؟ 1-** نعم **2-** لا
2. **كم واجهت من الصعوبة خلال اتبعاك الحمية الموصى بها؟
   1-** لا صعوبات **2-** قليلاً **3-** وسط **4-** كثيراً **5-** لم استطع الالتزام بالحمية الغذائية
3. **ما نوع الصعوبات التي واجهتها خلال محاولتك الالتزام بالحمية الغذائية؟
   1-** لا صعوبات **2-** لم أكن انوي التحكم في الطعام الذي ارغب بتناوله
   **3-** لم يكن باستطاعتي تجنب بعض أنواع الطعام الذي ليس من المفترض أن أتناوله
   **4-** لست افهم أي نوع من الحمية يجب إتباعها **5-** أخرى ــــــــــــــــــــــــــــــ
4. **خلال الأسبوع المنصرم, كم مرة اتبعت حميتك الغذائية الموصى بها؟
   1-**  كل الوقت **2-** اغلب الوقت **3-** نصف الوقت **4-** نادراً **5-** ولا مرة
